# Predictive maintenance and safety operation by device integration on the QUEST large experimental device

**DOI:** 10.1016/j.heliyon.2020.e04214

**Published:** 2020-06-24

**Authors:** Makoto Hasegawa, Kazuaki Hanada, Hiroshi Idei, Shoji Kawasaki, Takahiro Nagata, Ryuya Ikezoe, Takumi Onchi, Kengoh Kuroda, Aki Higashijima

**Affiliations:** Research Institute for Applied Mechanics, Kyushu University, Kasuga, Fukuoka, Japan

**Keywords:** Computer science, Electrical engineering, Safety engineering, Internet of things, System diagnostics, Electric power transmission, System fault detection, Computer-aided engineering, Predictive maintenance, IoT, Safety operation, FPGA, QUEST

## Abstract

As technology has improved in recent years, it has become possible to create new valuable functions by combining various devices and sensors in a network. This concept is referred to as the Internet of Things (IoT), and predictive maintenance is a new valuable function associated with the IoT. In large-scale experimental facilities with many researchers, it is not desirable that experiments cannot be performed due to sudden failure of equipment. For this reason, it is important to predict the failure in advance based on the measurement results of sensors and to perform repairs in a planned manner. On the Q-shu University experiment with steady-state spherical tokamak (QUEST) large experimental device, it is necessary to drive a large current of 50 kA, and the diagnosis of its power line deterioration is well performed as predictive maintenance through the evaluation of its contact resistances of several micro ohms order on the network. In addition, as an example of the IoT, mechanisms to assist safe operation, such as a sound alarm system and an entrance management system, are built by sharing experimental information between devices via the network.

## 1 Introduction

Predictive maintenance has become an effective approach for the maintenance of devices [1, 2] based on the concept that problems have to be anticipated and eliminated before they occur. These goals can be accomplished through the “Internet of Tings (IoT)” [3, 4]. Thus, various devices and sensors are connected to the network and communicate with each other in order to enhance the value of each device, and new services or functions can be created by these combinations of devices and sensors. By achieving predictive maintenance, sudden occurrences of problems can be suppressed and maintenance work can be executed in a planned manner. Thus, the reserve stock of repair parts and the labor costs to deal with sudden problems can be controlled, and will contribute the reduction of total costs.

**Figure 1:**
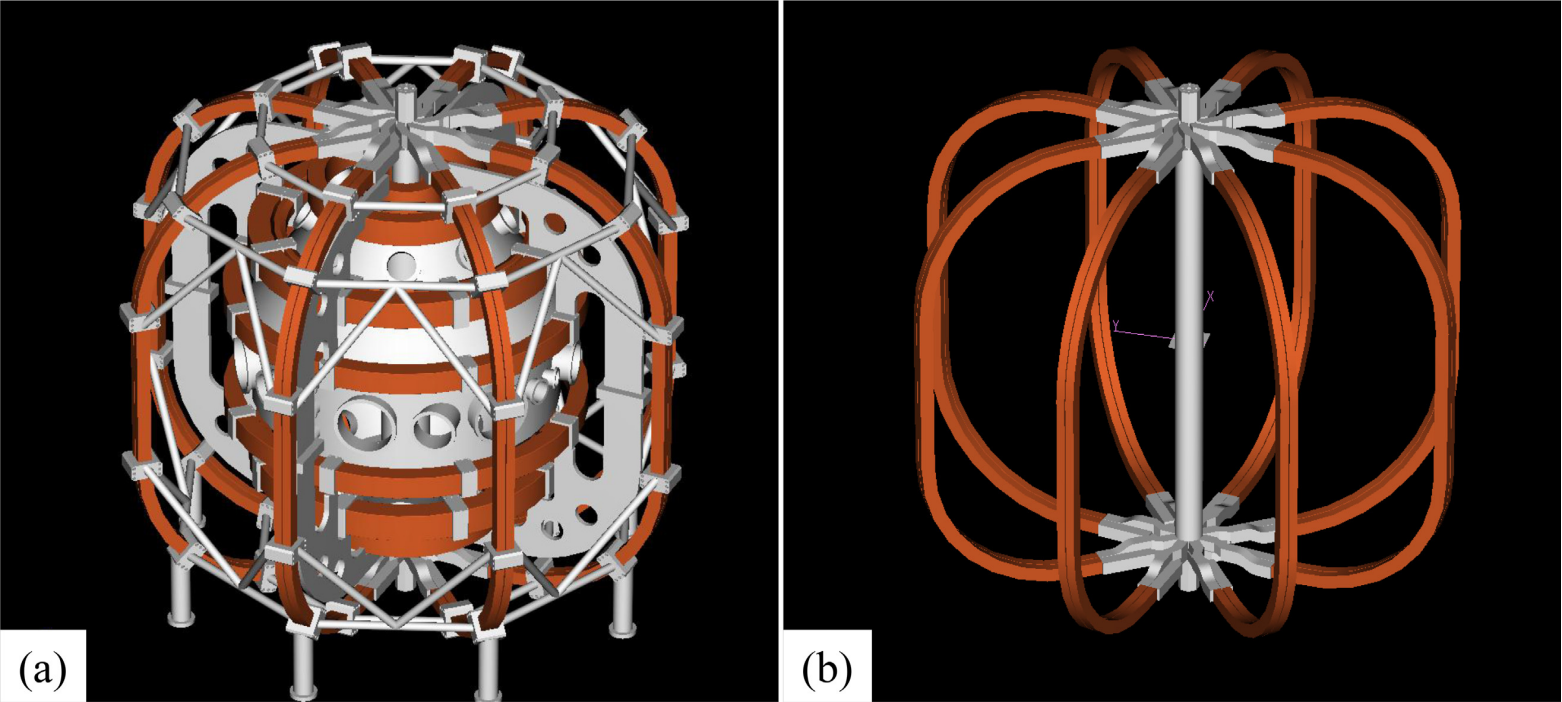
(a) Overall view of QUEST. Coils for confining plasma are installed around the vacuum vessel. (b) TF coil to generate a circular basic magnetic field.

The QUEST (Q-shu University experiment with steady-state spherical tokamak) has been built at the Advanced Fusion Research Center of Kyushu University, Japan [5]. Using QUEST (Figure 1(a)), steady state operation of plasma discharge [6, 7] and current drives by high frequency [8] are being studied, and many researchers from various facilities and organizations can visit the site and join experiments. For this reason, the situation whereby experiments cannot be performed due to device failure must be avoided. Hence, the concept of the predictive maintenance is important. Furthermore, it is also important to conduct operations safely in experiments involving many people.

The reminder of the present paper is organized as follows. Section 2 describe the configuration of overall control system of QUEST including networks. Section 3 describe the development of the diagnosis of power line deterioration as an example of predictive maintenance with the IoT. Section 4 describe the development of other peripheral sub-systems for safe operation with the IoT. Finally, our conclusions are presented in Section 5.

## 2 Overall configuration for control

The network used for the experiment involving QUEST is composed of two networks: an experimental network and a control network. The experimental network is used by many researchers, and various devices such as personal computers and measuring instruments are connected to this network. In this network, although some devices are registered to have static Internet Protocol (IP) addresses, other devices can be connected easily by dynamic IP addresses provided by a Dynamic Host Configuration Protocol (DHCP) server. On the other hand, in the control network, all of the devices are registered to have static IP addresses, and the table of Media Access Control (MAC) addresses and IP addresses is managed strictly to avoid wasting of network resources. On the boundary between the experimental network and the control network, a network router configured by a Linux server is installed. This server provides appropriate services, such as a common storage service to both networks, and controls the communication between these networks via a firewall, for example.

**Figure 2:**
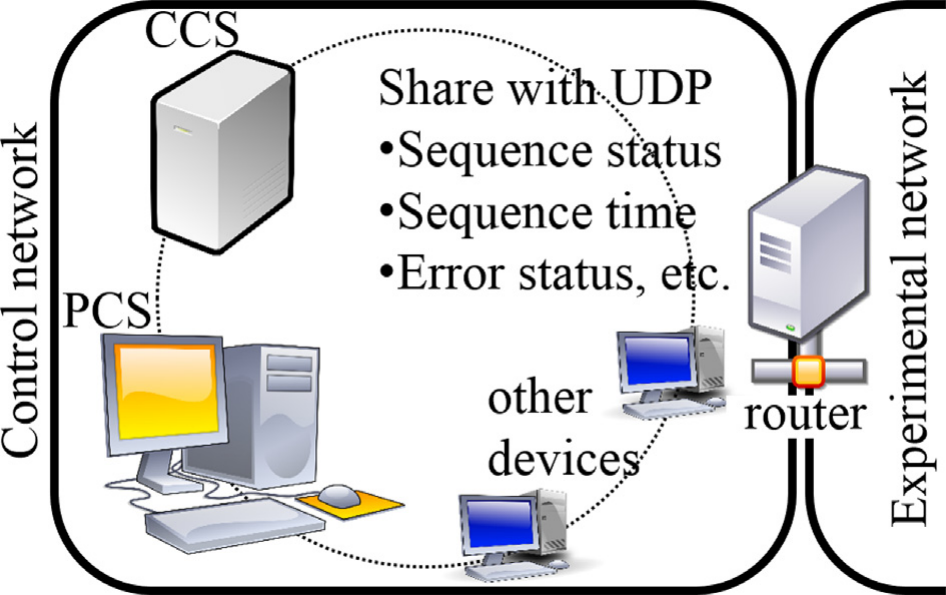
Overall configuration of the control network on QUEST with a central control system (CCS) and a plasma control system (PCS).

The overall control configuration for experiments is shown in Figure 2. The central control system (CCS) and the plasma control system (PCS) are connected to the control network. The CCS is composed of a PCI eXtensions for Instrumentation (PXI) system manufactured by National Instruments Corporation, and its programming language is LabVIEW, which is known as a graphical programming language. The CCS monitors peripheral subsystems, including the PCS, independent of whether their statuses are normal, and the CCS administrates the sequence of plasma discharges. When abnormal states are found on subsystems, the sequence is stopped or an alarm is sent to operators corresponding to the severity of the state. The CCS also provides information on sequences to all devices connected to the control network, such as the plasma discharge number, the status of the sequence, and the time of the sequence.

In the control network, communication between devices is performed using User Datagram Protocol (UDP). Simple text data that contains linefeed code and tab code as delimiters to separate data types and data values are sent to other devices via UDP in the control network. By adopting simple text-based communication with UDP, numerous types of devices can communicate with each other, even though their programming languages are different. Another advantage of using UDP is the ability to transfer data by broadcasting or multicasting. Thus, information concerning the sequence from the CCS is sent to other devices by broadcasting.

The PCS is also composed of a PXI Express (PXIe) system manufactured by National Instruments Corporation. During plasma discharge, the PCS acquires data, such as coil currents, magnetic signals, and visible lights of plasma, and analyzes the status of the plasma in real time. Then, the PCS sends control signals to other subsystems, such as coil power supplies, high-frequency oscillators, and gas fueling devices, for the real-time control of plasma. When the PCS detects the abnormal state of a device, the PCS terminates its plasma discharge and informs the CCS of the abnormal state by means of UDP.

## 3 Predictive maintenance

**Figure 3:**
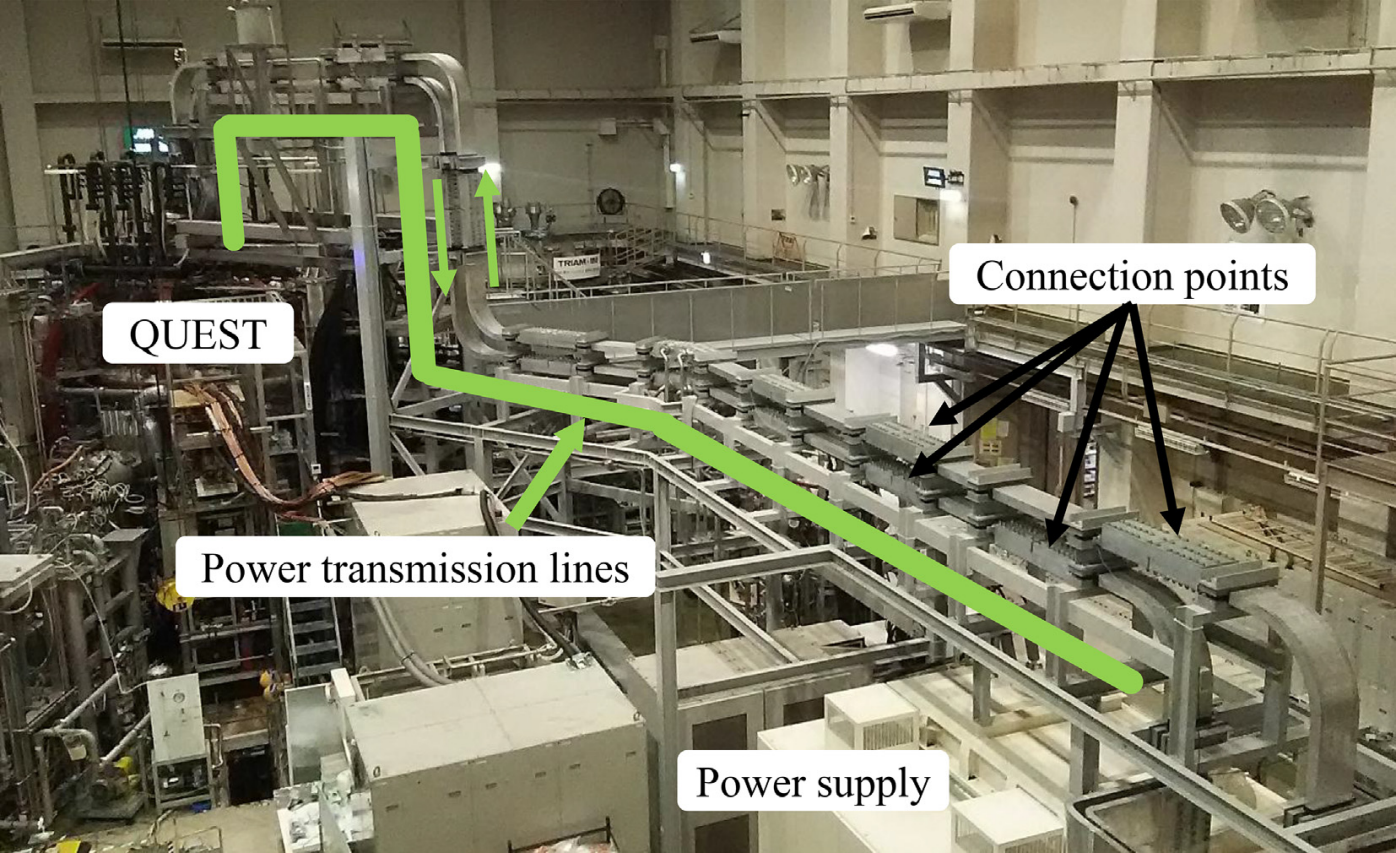
Transmission lines from power supply to QUEST. Outgoing and returning transmission lines with 16 connection points in total are installed in parallel.

For the plasma production and its confinement in a vacuum vessel, large currents must be conducted in the coils in order to produce magnetic fields, and cooling water must be circulated in order to remove the heat of the coils generated by the large currents. In QUEST, a power supply of 50 kA with a steady state is connected to a fundamental coil, referred to as a toroidal field (TF) coil, which generates a primary magnetic field of 0.25 T in order to confine the plasma (Figure 1(b)). The power transmission line of the coil is made of aluminum, rather than copper, in order to reduce the material costs and weight. This conductor is 300 mm in width and 80 mm in height, and has two passes of 40 mm in diameter for cooling water. The power transmission line is constructed by connecting these conductors and has 16 connection points (Figure 3).

Since the ionization tendency of aluminum is higher than that of copper or iron, electric erosion tends to occur on the surface, where the cooling water flows, because of the ionization of aluminum. In particular, when the electric erosion occurs at the contacting surface, the erosion causes not only an increase in the electrical contact resistance but also leakage of cooling water, and a serious failure involving other devices may occur. Thus, the periodic maintenance (polishing) of the contacting surface must be performed to make it flat. However, until now, there was no indication as to how often maintenance should be performed because the deterioration of the contacting surface was unknown and unpredictable. Therefore, in order to obtain this information, the measurements of contact resistances are considered because erosion of the conducting surface appears to cause an increase in contact resistance. The overall resistance of the power transmission line is designed and manufactured to be less than 100μΩ in order to reduce the power loss of the transmission line. Since there are 16 connection points on the power transmission line, each contact resistance will be several micro-ohms, values too low to be measured by commonly used multimeters. On the other hand, the maximum current flowing in this connection point is 50 kA, and the potential difference at this current and resistance is considered to become approximately several tens of millivolts. Thus, the contact resistances can be evaluated by measuring the potential differences during the usual usage of the TF coil power supply.

**Figure 4:**
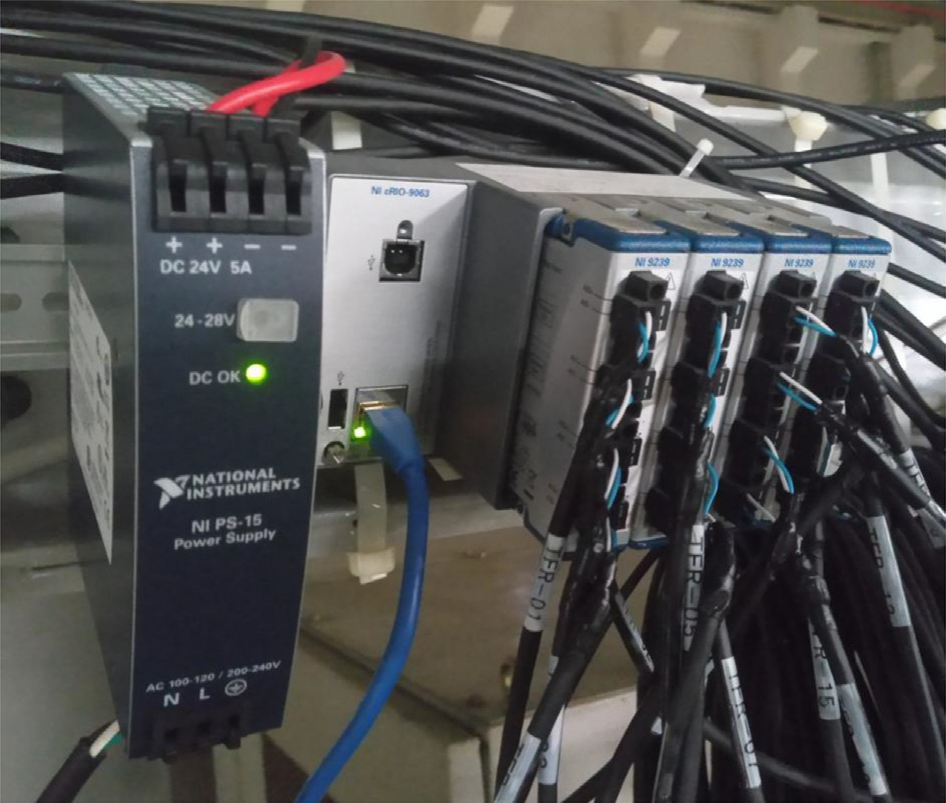
Picture of TFvm. Four NI 9239 modules are installed on cRIO controller.

**Figure 5:**
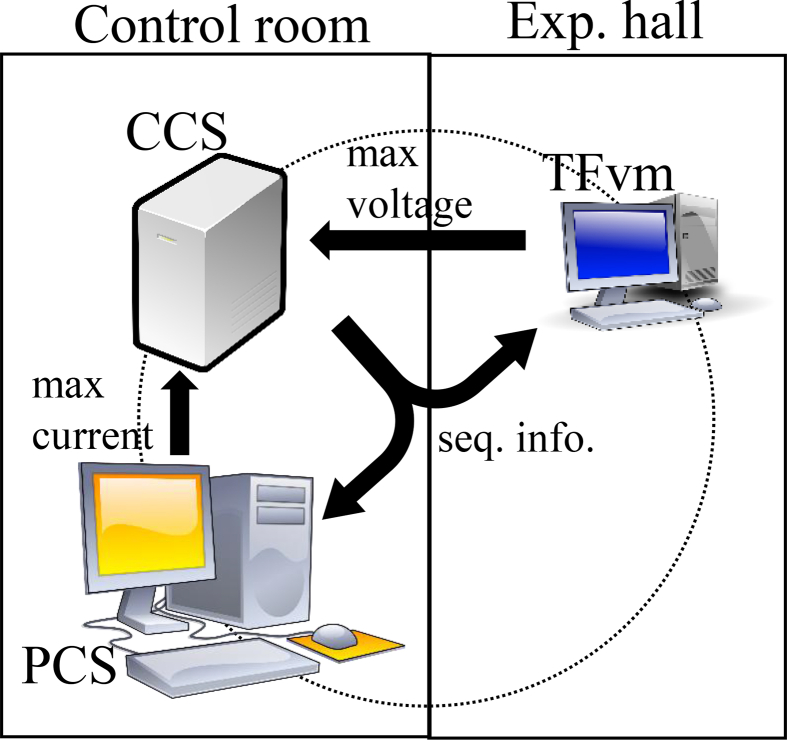
Diagram of calculation for power line resistances with a CCS, a PCS, and a TF coil voltage monitor (TFvm).

**Figure 6:**
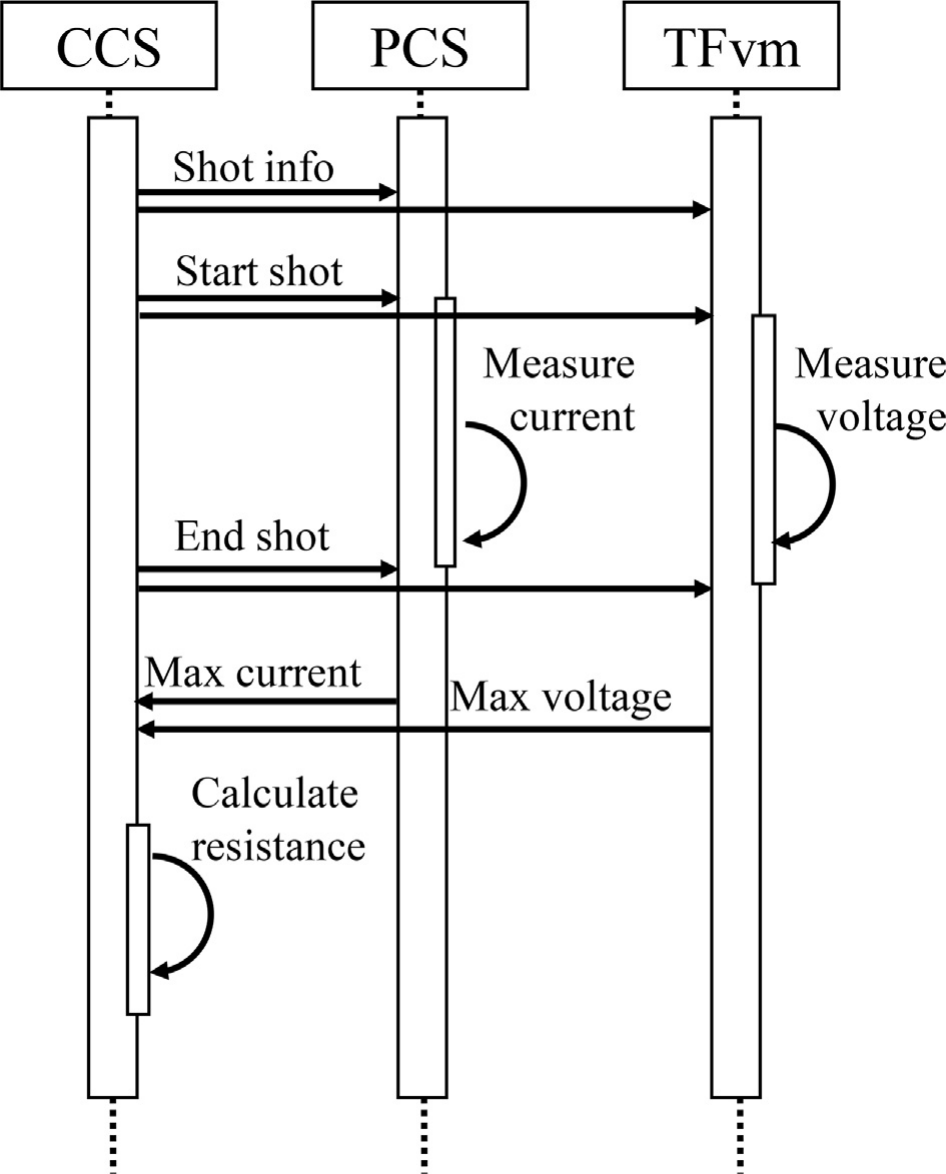
Sequence diagram to calculate resistances.

The potential differences are measured using a TF coil voltage monitor (TFvm), which uses a CompactRIO (cRIO) controller (National Instruments Corporation) including a field programmable gate array (FPGA). A FPGA is an integrated circuit, and the term “field programmable” means that a user or a customer can configure the integrated circuit after manufacturing using a hardware description language (HDL) [9]. The measurement modules of NI 9239, having specification of a voltage range of ±10 V, a sampling rate of 50 k samples/s/channel, and four channels with channel-to-channel isolation, are adopted (Figure 4). The voltage resolution of 24 bits is sufficiently high to measure several millivolt connection points. These four modules of NI 9239 are installed in the cRIO controller (cRIO-9063), and the voltages of 16 connection points are measured. The overall configuration and the sequence diagram are shown in Figure 5 and Figure 6, respectively. The CCS distributes the information of the discharge sequence with UDP. Receiving this information, the PCS drives the TF coil current and simultaneously records its maximum current. The TFvm also receives this information and records the maximum voltage of the 16 connection points during the discharge sequence. After the end of the sequence, the PCS and the TFvm send the maximum TF coil current and the maximum voltage, respectively, to the CCS with the UDP, and the CCS calculates and records the resistances of 16 connection points with this information. By measuring the resistances on a daily basis and storing their history, the tendency for an abnormality to occur becomes easier to identify.

In a typical discharge sequence of QUEST, because the plasma discharge starts after t = 0 s, the TF coil current starts from approximately -10 s, and is increased up to the target current, taking several seconds before t = 0 s. The current is thereafter typically kept constant during the plasma discharge, and the current is finally decreased to zero, taking several seconds. Thus, the time scales of the voltages measured by the TFvm should be several seconds.

**Figure 7:**
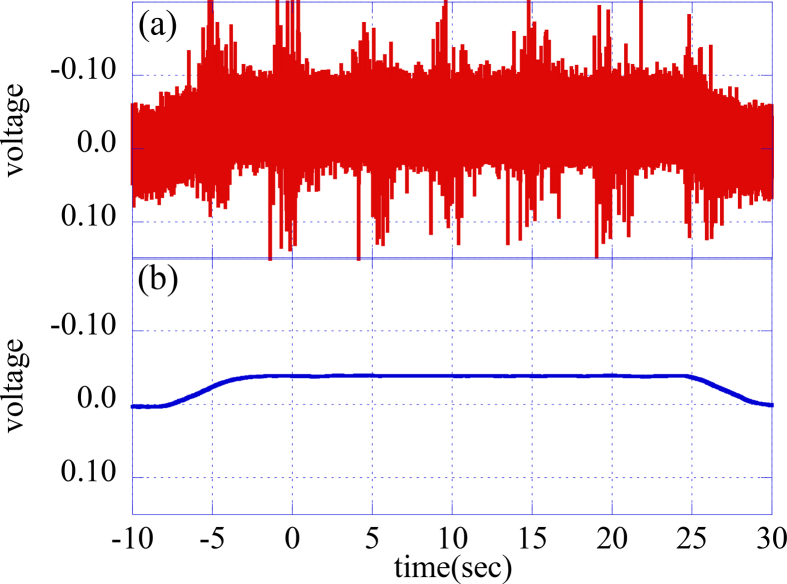
(a) Raw voltage signal of a connection point and (b) filtered voltage signal with a one-second low-pass filter.

An actual raw waveform of the voltage at one connection point is shown in Figure 7(a). This waveform is not smooth, but noisy, as compared to the corresponding TF coil current. The voltage measurement is considered to have been affected by noise from other devices, such as nearby high-power supplies and high-power and high-frequency oscillators in the experimental hall. Thus, the adoption of a digital low-pass filter with a FPGA is considered to remove the noise component. A low-pass filter without passive or active electronic components, but having a FPGA, has a simple overall configuration, and this configuration facilitates changing specifications, such as time constants. The first-order digital low-pass filter can be described by the number sequence given by:

(1) $$b_{n}=\left(a_{n}-b_{n-1}\right)\left(1-\exp\left(-\tau_{s}/\tau_{c}\right)\right)+b_{n-1}$$

where $a_{n}$ and $b_{n}$ are the number sequence of the measured and filtered voltages, respectively.

Since the sampling period $\tau_{s}$ is much smaller than the time constant $\tau_{c}$ of the low-pass filter in general, the number sequence of the filtered voltage $b_{n}$ is approximated as follows.

(2) $$b_{n}\approx \left(\tau_{s}/\tau_{c}\right)\left(a_{n}-b_{n-1}\right)+b_{n-1}$$

**Figure 8:**
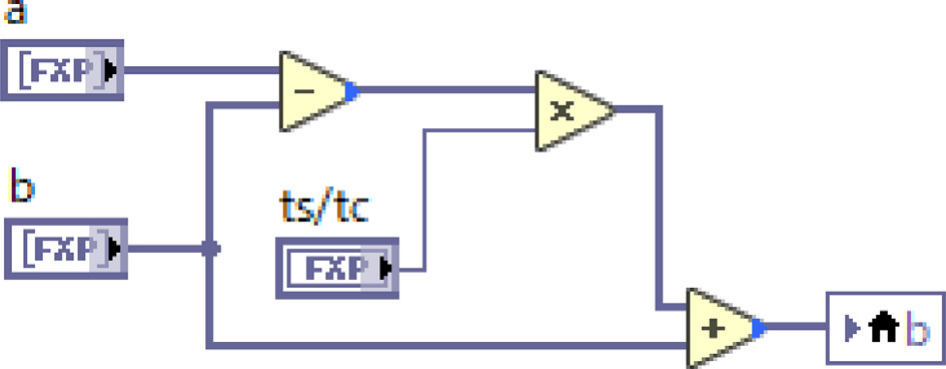
Typical low-pass filter written in LabVIEW FPGA. The variables a and b are fixed-point arrays with a number of elements equal to the number of 16 input channels.

In actuality, the condition of $\tau_{s}\ll \tau_{c}$ is sufficiently satisfied for the case in which $\tau_{s}$ = 40 μs and $\tau_{c}$ = 1 s. This equation can be represented with the LabVIEW graphical programming language by placing and connecting icons by wires (Figure 8), and can be compiled for execution on a FPGA. The filtered waveform is shown in Figure 7(b). Since the high-frequency noise is removed by the low-pass filter with a sampling frequency of 25 kHz, a smooth waveform similar to the TF coil current can be delivered in real time. The maximum voltages of filtered waveforms of 16 connection points are monitored continuously and are sent to the CCS after the discharge sequence. The PCS also calculates the low-pass filtered waveform of the TF coil current in real time with same time constant of 1 s and monitors its maximum value during the sequence. After the sequence, the CCS calculates the resistances of 16 connection points with the values of the maximum voltages from the TFvm and the maximum current from the PCS and records these values in order to determine the hysteresis.

**Figure 9:**
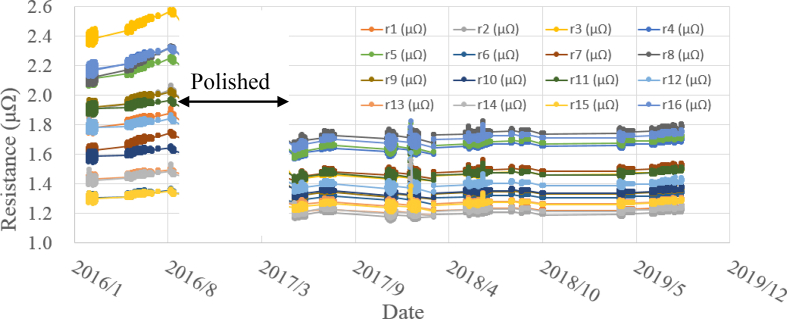
History of evaluated resistances of 16 connection points.

The time history of the resistance of 16 connection points is shown in Figure 9. Since these resistances were gradually increased until August 2016, the electric erosion was judged to be progressing. Thus, the 16 contacting surfaces were polished flat in order to reduce the resistances by March 2017. After polishing, all 16 resistances were decreased and generally remained constant until August 2019. These resistances gradually increase when monitored over a long period. The progress of the electric erosion could be evaluated from absolute increases and change rates. Thus, problems such as water leakage can be predicted and prevented in advance by linking multiple devices, such as CCS, PCS, and TFvm devices, together to monitor the increase of contact resistances. This new creation of valuable functions by linking devices each other is an example of the IoT and is helpful for predictive maintenance.

## 4 Systems for safe operation

The experimental hall of QUEST is a restricted area during plasma discharge in order to avoid damaging human health due to X-ray emission or the emission of electromagnetic waves. However, researchers often enter the experimental hall during the interval of plasma discharges to adjust their measurement instruments. Thus, it is necessary to carefully perform plasma discharges after confirming that no human is in the experimental hall. In order to support this confirmation, two mechanisms are implemented as examples of the IoT to create new valuable functions by linking devices: the entrance management system (EMS) or the sound alarm system (SAS).

**Figure 10:**
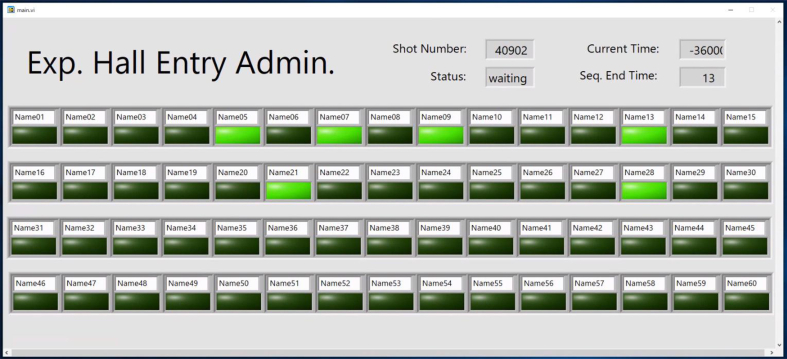
Interface of the entrance management system provided by LabVIEW on a touch display. Bright buttons indicate persons in the experimental hall.

**Figure 11:**
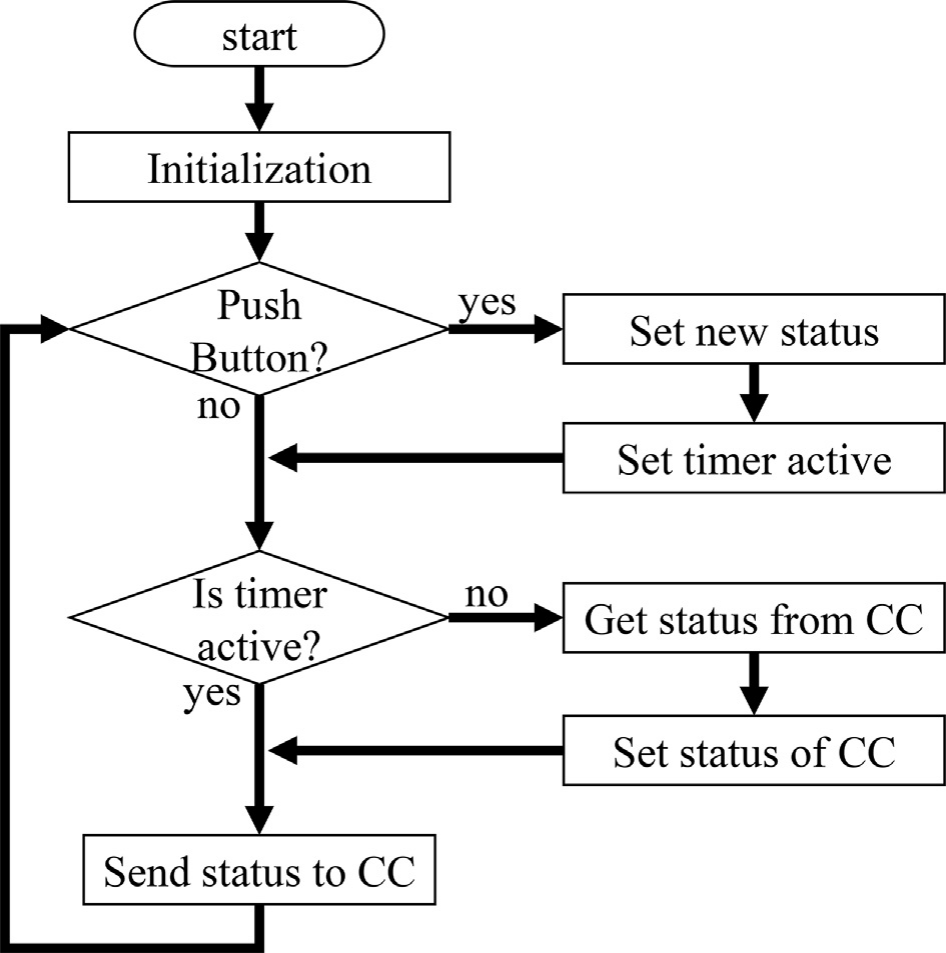
Flow chart of one peripheral for the entrance management system (EMS).

The EMS includes the CCS and other peripherals, and peripherals are installed near each entrance of the experimental hall. One peripheral is composed of a touch display and a mini PC having dimension of 113 mm × 38 mm × 12 mm, which can communicate with the CCS via Wi-Fi. The runtime environment of LabVIEW is installed on the mini PC, and a user interface of buttons with the names of researchers created using LabVIEW is displayed on the touch display (Figure 10). Researchers have to turn on or off the button labeled with their own names upon entering or exiting the experimental hall. Since the statuses of these buttons are shared with other peripherals via the CCS with UDP, the current entry status can be checked at other places, and the buttons can be turned on or off at other locations. Since the entry status is also shared with the CCS, the CCS can stop the discharge sequence when someone is in the experimental hall. A flow chart of one peripheral is shown in Figure 11. When the button is pushed, a timer of several seconds is activated to ignore the status indicated by the CC. The CC receives the new status of the peripheral and broadcasts this status to all other peripherals. If the peripheral's timer is not active, it receives the status from the CC.

In order to facilitate maintenance of the EMS, the executable program LabVIEW is stored on common storage, and each peripheral executes LabVIEW on its terminals. The name list of researchers is also stored in common storage, and each peripheral reads that list at the initialization of the program. As a result, when the program or the name list is modified, changes need only be made at one place. Although an executable program is commonly used, this program can also be operated differently at each peripheral by judging the IP addresses of the mini PCs. For example, each peripheral sends its information to a different receiving UDP port of the CCS.

The SAS also includes the CCS and other terminals that can produce sound with their speakers. Since researchers enter the experimental hall to make adjustments of their measurement instruments during the interval of plasma discharges, researchers have to continuously monitor the status of the sequence in order to exit the experimental hall before the plasma discharges start. The SAS helps researchers to know the status of the sequence through several kinds of sounds at terminals. Receiving the status of the sequence with UDP from the CCS, the terminals play music 2 min before plasma discharge and sound an alarm and a chime 1 min and 10 s, respectively, before plasma discharge. These terminals also sound warning alarms notifying researchers of abnormal status from the CCS. Since general desktop PCs or laptop PCs can be used as terminals, the installation location and number of installations can be changed freely. In QUEST, these terminals are installed in the control room and the experimental hall in order to enable researchers to call for attentions. Thus, although the EMS and the SAS are composed of simple touch panels, mini PCs, and general PCs, new valuable functions, such as entry management and acoustic alarms, can be produced by connecting simple devices.

## 5 Conclusion

Diagnostics for the deterioration of the TF power transmission line have been developed as a method of predictive maintenance. These diagnostics are very effective and will help to reduce operational costs by reducing the risk of sudden failures. This is one example of the IoT, which can create new benefits by network communication between various devices and sensors. An entry management system and a sound alarm system have also been developed as examples of IoT systems. These systems, which are composed of simple equipment, are very useful and assist in the safe operation of QUEST. In the future, the data of many measurement instruments, such as vacuum gauges, flowmeters, and temperature gauges, installed at various locations will be shared via network communication at QUEST, and new benefits to assist in predictive maintenance and safe operations will be created more frequently.

## Declarations

### Author contribution statement

Makoto Hasegawa: Conceived and designed the experiments; Performed the experiments; Analyzed and interpreted the data; Contributed reagents, materials, analysis tools or data; Wrote the paper.

Kazuaki Hanada, Hiroshi Idei, Ryuya Ikezoe, Kengoh Kuroda & Aki Higashijima: Conceived and designed the experiments.

Shoji Kawasaki, Takahiro Nagata & Takumi Onchi: Conceived and designed the experiments; Performed the experiments; Contributed reagents, materials, analysis tools or data.

### Funding statement

This work was supported by a grant from the NIFS Collaboration Research Program (NIFS17KUTR122).

### Competing interest statement

The authors declare no conflict of interest.

### Additional information

No additional information is available for this paper.
